# Understanding Alcohol Abuse in Deceased Donors: Effects on Liver Transplant Results

**DOI:** 10.3390/jcm14082773

**Published:** 2025-04-17

**Authors:** Agata Konieczka, Oskar Kornasiewicz, Michal Skalski, Joanna Raszeja-Wyszomirska, Michał Grąt

**Affiliations:** 1 Department of General, Transplantation and Liver Surgery, UCC MUW, 02-977 Warsaw, Poland; agata.anna.konieczka@gmail.com (A.K.); michal.skalski1@wum.edu.pl (M.S.); michal.grat@wum.edu.pl (M.G.); 2 Liver and Internal Medicine Unit, Department of General, Transplant and Liver Surgery, Medical University of Warsaw, 02-097 Warsaw, Poland; joanna.wyszomirska@wum.edu.pl

**Keywords:** extended-criteria donor, liver transplantation, alcohol abuse

## Abstract

**Background/Objectives:** Liver transplantation is a life-saving procedure for patients with end-stage liver disease. In recent years, the demand for liver transplantation has surpassed the supply of available donor organs. Utilizing extended-criteria donors (ECDs) alleviates the scarcity of suitable donor livers for transplantation. One of the ECD was donors with a history of alcohol abuse. Liver grafts from donors with a history of chronic and active alcohol abuse are typically promptly excluded, diminishing the available organ pool. This highlights the need to re-evaluate the donor exclusion criteria and expand the organ pool to address the ongoing shortage. **Methods:** We examined adult (>18 years) liver transplant recipients who received deceased donor livers and had a documented history of alcohol abuse between 2011 and 2024. Liver transplant indications were conventional and included hepatitis C virus (HCV), non-alcoholic steatohepatitis, alcoholic liver disease, alcoholic liver disease coexisting with HCV, cryptogenic cirrhosis, chronic cholestatic liver disease, primary biliary cholangitis, primary sclerosing cholangitis, metabolic liver disease, hepatocellular carcinoma, and alcoholic hepatitis. We compared the 1-year, 5-year, and 9-year survival rates with those of liver recipients from non-alcohol-consuming donors. **Results:** In total, 370 liver recipients from deceased donors with a documented history of alcohol abuse were included. At 1 year post-transplant, survival was comparable between the two groups. **Conclusions:** Liver transplantation from deceased donors with a history of alcohol abuse yielded survival rates and liver function outcomes comparable to those from non-alcohol-using donors. By expanding the criteria to include carefully screened alcohol-using donors, transplant programs can improve access to life-saving transplantations.

## 1. Introduction

Liver transplantation is widely recognized as the definitive treatment for patients with end-stage liver disease (ESLD), offering a potential cure and significantly improving survival and quality of life [1]. Despite the life-saving potential of this procedure, the demand for liver transplants far exceeds the availability of suitable donor organs, creating a critical bottleneck in the treatment of liver diseases worldwide [2]. Organ shortage has driven the transplantation community to explore and implement strategies to expand the donor pool, including the use of extended-criteria donors (ECDs).

ECDs refer to donor livers that may not meet conventional standards because of factors such as older donor age, prolonged cold ischemia time, presence of metabolic abnormalities, or history of disease that could affect liver function [3]. These criteria are broader than those traditionally accepted, with the goal of increasing the number of transplantable organs while carefully managing the associated risks. The most common ECDs include donor’s age >65 years, donor steatosis, donors after cardiac death, positive (HCV+) donors, positive (HIV+) donors, and donors with history of alcohol abuse [3]. Despite concerns about potential complications, ECD liver transplantation has become an increasingly common practice, given the pressing need to address organ shortage [4].

The organ shortage has led to taking into consideration livers from donors with a history of alcohol abuse. Chronic alcohol consumption is a major risk factor for liver disease, and organs from individuals with a history of significant alcohol consumption are often excluded from transplantation because of concerns about compromised liver function and poor post-transplant outcomes [5]. However, recent research involving large cohorts in the US and Europe demonstrated that while slightly higher risks of late mortality were observed in recipients of grafts from donors with a history of alcohol abuse, these risks are typically minor and can be mitigated through rigorous pre-transplant screening [6]. Exclusion of these organs further exacerbates organ scarcity, especially in countries like Poland, where alcohol consumption is prevalent and the number of individuals with alcohol abuse disorders is significant. Lack of anti-promotional alcohol policies and relatively low alcohol prices in Poland in comparison with Europe has led to an increase in the Polish drinking population by 35%. Moreover, according to available data, around 30,000 deaths in Poland in 2021 were related with alcohol consumption [7]. Current guidelines lack specific recommendations for assessing the suitability of livers from donors with a history of alcohol use, leading to variability in clinical practice and the potential to overlook organs that may be viable for transplantation [8].

This study aimed to address this gap by evaluating the outcomes of liver transplantation using organs from deceased donors with documented histories of alcohol abuse. By comparing the survival rates of recipients of these livers with those of non-alcohol-using donors, this study aimed to inform the development of nuanced guidelines for donor selection, potentially expanding the donor pool and improving access to life-saving liver transplants.

## 2. Materials and Methods

This retrospective cohort study analyzed adult liver transplant recipients (aged > 18 years) who underwent transplantation between January 2011 and September 2024. The inclusion and exclusion criteria were defined in Table 1. This study focused on recipients who received livers from deceased donors and had documented histories of alcohol abuse. Alcohol abuse was defined according to data from medical records, including a documented history of alcohol consumption provided by family, social services, hospital history, and general practitioners’ outpatient clinics. The study did not distinguish between occasional and chronic heavy drinkers, since in many cases there was insufficient data to divide them into separate groups. Liver recipients from donors who did not consume alcohol during the same period were included in the control group. All transplants were performed at the Warsaw Medical University Teaching Hospital.

Donors were classified as having a history of alcohol abuse if they had documented chronic alcohol consumption, with or without clinical signs of liver injury. The organ viability was confirmed during the perioperative assessment. In the case of any warranties, histopathological examination of liver surgical biopsy was performed. Indications for transplantation included hepatitis C virus (HCV) infection, non-alcoholic steatohepatitis (NASH), alcoholic liver disease (ALD), ALD coexisting with HCV, cryptogenic cirrhosis, chronic cholestatic liver disease, primary biliary cholangitis, primary sclerosing cholangitis, metabolic liver disease, hepatocellular carcinoma, and alcoholic hepatitis. The primary outcome measure was recipient survival at 1, 5, and 9 years post-transplant. Secondary outcomes included graft survival and liver function test results during follow-up. Survival rates for recipients of livers from alcohol-abusing donors were compared with those from non-alcohol-using donors using Kaplan–Meier survival analysis. Log-rank tests were used to assess the statistical significance of the differences in survival curves between the two groups. Cox proportional hazard regression models were used to adjust for potential confounders, including recipient age, sex, Model for End-Stage Liver Disease (MELD) score, and donor ECD status.

Continuous variables were summarized using means and standard deviations or medians and interquartile ranges, depending on their distribution. Categorical variables were expressed as frequencies and percentages. Statistical analyses were performed in R Studio using the R programming language. The groups were selected from a pool of 3090 patients by propensity score matching with an allocation ratio of 2:1. This study aimed to perform a balanced comparison between groups by matching individuals by age. Patients were selected according to the indications for liver transplantation (ALD, primary sclerosing cholangitis, echinococcosis, or primary biliary cirrhosis), and recipients who received a liver transplant that met the ECD were included. Duplicates were filtered to exclude individuals who underwent liver re-transplantation.

All statistical analyses were performed using R language via RStudio Team (2020). (RStudio: Integrated Development for R. studio, PBC, Bostron, MA, USA, URL http://rstudio.com/) (R version 4.3.1 (16 June 2023)).

The United Network for Organ Sharing (UNOS) defines alcohol abuse in donors as chronic consumption that significantly affects health, particularly in terms of clinical signs of organ damage or dysfunction. In contrast, the criteria used in this study were broader and encompassed reports from family members, social services, and outpatient clinic histories, reflecting the sociocultural context of Poland. Unlike the standardized UNOS definition, the Polish approach acknowledges the difficulty in objectively quantifying alcohol consumption, especially in a society where moderate or social drinking is commonplace and not necessarily problematic. This broader classification helps capture a wider range of potential donors without excluding viable organs solely because of ambiguities in alcohol use history.

## 3. Results

This study analyzed the outcomes of liver transplants performed between 2011 and 2024 in recipients of livers from deceased donors with a documented history of alcohol abuse compared with recipients of livers from donors without a history of alcohol use. The primary outcome was post-transplant survival at one, five, and nine years (Table 2).

Table 3 provides the demographic characteristics of the liver transplant recipients. No statistically significant difference was observed between recipients of livers from alcohol-abusing and non-alcohol-using donors in terms of age (median age, 52 years for both groups; *p* = 0.969), body mass index (BMI) (median, 24.67 vs. 25.31; *p* = 0.597), or MELD score at transplantation (median, 12; *p* = 0.924). However, the total ischemia time was longer in the alcohol-abusing donor group (mean, 572 min) than in the non-alcohol-using donor group (mean, 525 min; *p* = 0.168), indicating that the livers from alcohol-abusing donors experienced longer cold ischemia.

**Figure 1:**
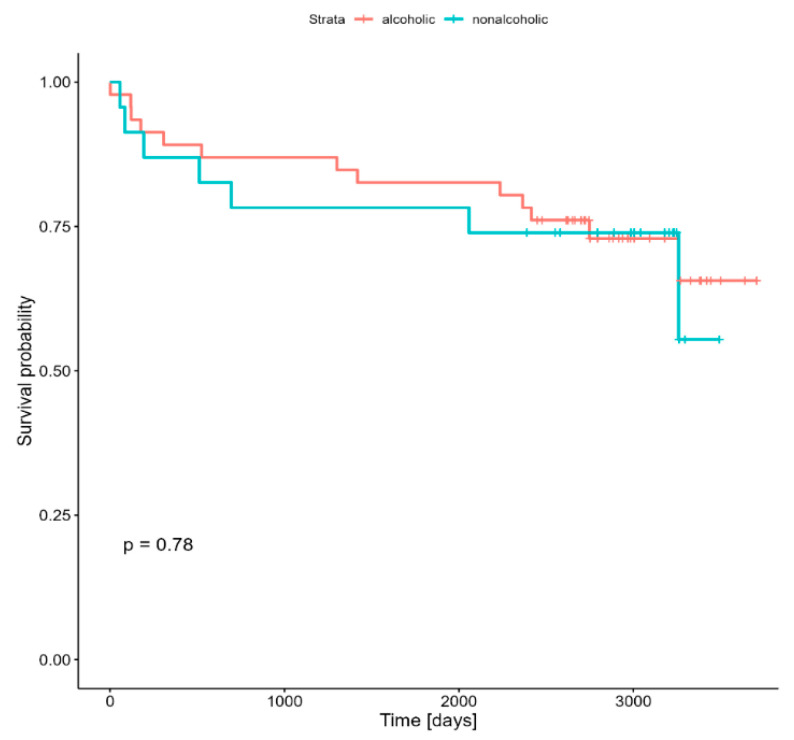
Long-term post-transplant survival of patients with ECD liver recipients and non-ECD liver recipients. ECD: extended-criteria donor.

Figure 1 shows the Kaplan–Meier survival curves comparing long-term post-transplant survival between recipients of livers from alcohol-abusing and non-alcohol-using donors. At 1 year post-transplant, survival was comparable between the two groups (87% vs. 89%, *p* > 0.05). Five-year survival showed no significant difference (78.3% vs. 82.6%, *p* > 0.05), and the 9-year survival rates of the groups were 61% vs. 55.4% (*p* > 0.05). These findings indicate that liver transplantation using organs from donors with alcohol abuse does not negatively affect the long-term survival of the recipients.

Additionally, the peak levels of liver function markers, including alanine aminotransferase (ALT), gamma-glutamyl transferase (GGTP), and alkaline phosphatase (ALP), showed no significant differences, indicating similar graft function post-transplant between the groups. Cox proportional hazards regression analysis, adjusted for recipient’s age, confirmed that the use of livers from alcohol-abusing donors was not associated with an increased risk of mortality (hazard ratio [HR] = 1.05, 95% confidence interval [CI] 0.94–1.18, *p* = 0.463).

## 4. Discussion

The results of this study suggest that liver transplants from deceased donors with a history of alcohol abuse can provide outcomes comparable to those of transplants from donors who did not use alcohol in terms of both short- and long-term survival. Notably, these findings challenge prior assumptions that alcohol-abusing donors might yield inferior outcomes, demonstrating instead that liver recipients from such donors exhibited similar 1-year, 5-year, and 9-year survival rates to recipients of organs from non-alcohol-using donors. Furthermore, no significant differences were observed in secondary outcomes such as graft survival or liver function. These findings support the use of livers from alcohol-abusing donors as a viable option for expanding the donor pool, particularly in regions such as Poland, where alcohol consumption is prevalent. This aligns with studies highlighting the importance of re-evaluating rigid donor exclusion criteria, although some earlier studies suggested potential risks associated with alcohol consumption history.

Livers from donors with a history of alcohol abuse displayed robust outcomes despite often having longer cold ischemia times than non-alcohol-abusing donor organs, demonstrating the resilience or “strength” of these grafts, as they were able to tolerate prolonged ischemia without compromising recipient survival or graft function. This resilience may be attributed to the liver’s regenerative capacity and the adaptive mechanisms that develop in response to alcohol exposure. The absence of significant differences in liver function markers or survival outcomes highlights that these livers are not inherently predisposed to poor outcomes as commonly feared.

Arora et al. [9] corroborate these observations, demonstrating that heavy alcohol use among deceased donors did not adversely impact post-transplant outcomes in recipients. Their study, which included an analysis of liver transplant recipients stratified by donor alcohol use history, reported no significant differences in recipient survival or graft failure rates. Similarly, data from studies examining the use of ECDs [4,10] highlight that expanding donor eligibility criteria, including the use of organs with certain risk factors, does not compromise recipient survival and can significantly reduce waitlist mortality by increasing the donor pool. For instance, the use of ECDs has been shown to decrease the waiting time for liver transplantation without negatively affecting post-transplant survival [4].

At our transplantation center, we did not disqualify organs from donors with a history of alcohol abuse. This approach was influenced by Poland’s sociocultural context, where alcohol consumption is a common and widely accepted practice. As emphasized by Correia et al. [11], alcohol consumption patterns in Europe are highly variable but often normalized within certain regions. Disqualifying such donors would significantly diminish the pool of available organs for transplantation, exacerbating the critical organ shortage. Recognizing that moderate or socially acceptable alcohol use does not necessarily equate to organ unsuitability, we aligned our practices with the broader goal of maximizing donor utilization.

One of the key challenges in using livers from alcohol-abusing donors is assessing the true extent of alcohol use and its impact on liver quality. Definitions of alcohol abuse vary widely, and in many cases, socially acceptable drinking habits, such as regular beer consumption, are not classified as problematic. This variability underscores the need for standardized protocols to assess alcohol use histories in potential donors. Biomarkers such as phosphatidylethanol, which has a sensitivity of 75% and a specificity of 97%, could be employed to measure alcohol misuse objectively; however, such tools are not yet routinely used in clinical practice. Moreover, as noted in donor assessments [12], a significant proportion of potential liver donors are discharged because of variable criteria or logistical issues rather than intrinsic organ quality. Reviewing and refining these discharge criteria can further enhance donor organ availability without compromising safety [12].

This study, supported by the findings of Mangus et al. [5] and Arora et al. [9], reinforces the decision not to divide donor groups based on the type or quantity of alcohol consumed. However, while this approach simplifies donor evaluation, potential limitations exist, such as the difficulty in accurately assessing long-term liver health in alcohol-abusing donors. Both studies observed that post-transplant outcomes were not significantly affected by variations in alcohol use history among donors. Dividing donor groups based on specific alcohol consumption patterns adds complexity to donor evaluation without providing meaningful prognostic value. Instead, a holistic evaluation of donors based on overall liver function ensures that viable organs are not unnecessarily excluded. This pragmatic approach reduces the risk of over-stratification, which could further limit the donor pool without improving transplantation outcomes.

In rare cases in which the history of alcohol abuse was significant or suspected to be severe, an intraoperative histopathological examination of the donor liver was conducted. This step serves as an additional safeguard to ensure organ quality. However, such examinations were infrequent, as the majority of donors with a history of alcohol use did not exhibit gross or histological evidence of advanced liver disease. This finding further supports the inclusion of donors in the transplantation pool without undue hesitation.

Donors with a history of alcohol abuse represent a valuable segment in the ECD pool. As the demand for liver transplants continues to outpace the supply, excluding this group solely based on alcohol use history would unnecessarily narrow the pool of available organs. The findings of this study, along with those of Arora et al. [9], Maggi et al. [13], and large-scale ECD-focused analyses [4,10], suggest that these donors can be safely included in transplantation programs, provided that they meet other standard selection criteria. By adopting this inclusive approach, transplant centers can increase the availability of life-saving organs for patients with ESLD.

Future research should focus on the long-term graft quality and function in recipients of alcohol-abusing donor livers. Investigating specific patterns of alcohol use, including the duration, severity, and type of alcohol consumed, may provide insights into their impact on transplantation outcomes. However, given the evidence from Mangus et al. [5], Arora et al. [8], and the current study, dividing donors based on alcohol type or amount is currently not justified. Additionally, developing and implementing objective measures such as biomarkers or standardized alcohol use assessment tools [14] will enhance donor evaluation protocols and ensure the continued safety and efficacy of transplantation practices.

## 5. Conclusions

The results of this study highlighted the potential of livers from donors with a history of alcohol abuse for achieving outcomes comparable to those from donors without alcohol use. The resilience of these organs, even in the context of longer cold ischemia times, challenges the traditional exclusion criteria and supports their inclusion in the extended-criteria donor pool. By integrating these findings into clinical practice, transplant programs can expand the donor pool, improve access to life-saving transplants, and address persistent organ shortages without compromising recipient outcomes. However, further research is needed to assess the long-term impact of using livers from alcohol-abusing donors, particularly regarding graft durability, potential late-onset complications, and the influence of different alcohol consumption patterns on transplant success. Continued studies will help refine donor selection criteria and optimize transplantation protocols to ensure both safety and efficacy.

## Figures and Tables

**Table 1:** The characteristics of the groups (inclusion and exclusion criteria).

Inclusion Criteria	Exclusion Criteria
Liver transplants recipients ≥ 18 years of age	Donors without history of chronic alcohol consumption, i.e., single alcohol misuse was not classified as donors with history of alcohol abuse
Deceased liver donors with documented histories of alcohol abuse	Donors after circulatory death
Indications for transplantation: hepatitis C virus (HCV) infection, non-alcoholic steatohepatitis (NASH), alcoholic liver disease (ALD), ALD coexisting with HCV, cryptogenic cirrhosis, chronic cholestatic liver disease, primary biliary cholangitis, primary sclerosing cholangitis, metabolic liver disease, hepatocellular carcinoma, and alcoholic hepatitis	Liver recipients that underwent liver re-transplantation

**Table 2:** Comparison of survival rates between liver transplant recipients.

	Recipients of ECD Liver	Non-ECD Liver Recipients	
5-day survival	100.00% (100–100%)	97.80% (93.7–100%)	*p*-value 0.78
1-year survival	87.00% (74.2–100%)	89.10% (80.6–98.6%)	
5-year survival	87.00% (63.1–97.1%)	82.60% (72.4–94.3%)	
9-year survival	55.40% (29.9–100%)	65.60% (49.8–86.4%)	

ECD: extended-criteria donor.

**Table 3:** Demographical data for liver transplant recipients.

	Recipients of ECD Liver	Non-ECD Liver Recipients	*p*-Value
Age (years, median)	52	52	0.969
Body mass index (median)	24.67	25.31	0.597
Total ischemia time (mean)	572.174	525.261	0.168
MELD (median)	11	12	0.924
Peak alanine aminotransferase	144	609	0.684
Peak gamma-glutamyl transferase	1350	1350	0.177
Peak alkaline phosphatase	735	943	0.765

ECD: extended-criteria donor; MELD: Model for End-Stage Liver Disease.

## Data Availability

Data are contained within the article.

## Abbreviations

The following abbreviations are used in this manuscript:

ESLD	End-stage liver disease
ECD	Extended-criteria donor
HCV	Hepatitis C virus
NASH	Non-alcoholic steatohepatitis
UNOS	United Network for Organ Sharing
MELD	Model for End-Stage Liver Disease
ALD	Alcoholic liver disease
BMI	Body mass index
ALT	Alanine aminotransferase
GGTP	Gamma-glutamyl transferase
HR	Hazard ratio
ALP	Alkaline phosphatase
